# Supplementary material for: Prevalence and risk factors for severe food insecurity and poor food consumption during a drought emergency in Ethiopia
Source: PLOS Glob Public Health. 2025 Sep 11;5(9):e0004636. doi: 10.1371/journal.pgph.0004636 (PMC12425312; doi:10.1371/journal.pgph.0004636)
Supplement: S1 Table — This file provides definitions for each variable assessed in this study. (DOCX) [file pgph.0004636.s001.docx]

**S1 Table: Variable Definitions for *Prevalence and risk factors for severe food insecurity and poor food consumption during a drought emergency in Ethiopia***

| **Variables** | **Definitions** |  |
| --- | --- | --- |
|  |  |  |
| **Age Group** | Age group of the woman/girl of reproductive age (WRA) (15-49 years) that responded to the survey |  |
| WRA 20-29: Reference Group | Households where the woman/girl of reproductive age survey respondent was between 20 and 29 years of age. |  |
| WRA 15-19 | Households where the woman/girl of reproductive age survey respondent was between 15 and 19 years of age. |  |
| WRA 30-49 | Households where the woman/girl of reproductive age survey respondent was between 30 and 49 years of age. |  |
| **Number of CU5 in household** | The number of children under 5 years of age that resided within the household that was surveyed. |  |
| No CU5 in household: Reference Group | Households that did not have any children under 5 years of age residing within them. |  |
| One CU5 in household | Household that had one child under 5 years of age residing within it. |  |
| Two or more CU5 in household | Households that had 2 or more children residing within them. |  |
| **Demographics** |  |  |
| WRA Currently Pregnant | Households where the woman/girl of reproductive age respondent was pregnant at the time of data collection. |  |
| WRA History of Pregnancy | Households where the woman/girl of reproductive age respondent had a history of pregnancy |  |
| WRA Marital Status: Married | Households where the woman/girl of reproductive age respondent was married. |  |
| **Educational Level** |  |  |
| WRA No school | Households where the woman/girl of reproductive age survey respondent did not receive any formal education. |  |
| Household Head No school | Households where the household head did not receive any formal education  Household head refers to either the household’s woman/girl of reproductive age respondent if the woman/girl of reproductive age was the household head, or the individual the woman/girl of reproductive age respondent considered to be head of the household. The woman/girl of reproductive age respondent provided this information regardless of who was the head of household. |  |
| **Economic Activity** |  |  |
| WRA performed cash earning work last 12 months | Households where the woman/girl of reproductive age survey respondent performed cash-earning work in the past 12 months |  |
| Household saved money | Households that saved money |  |
| Household had daily per capita food consumption costs <1.61 USD | Households that consumed less than 1.61 USD of food per day on average. Indicator of food poverty based on the 2011 adjusted poverty line computed via consumer price index. |  |
| Household used financial services | Households that either used agricultural credit, had savings, and/or used crop insurance |  |
| Household had access to a plot of land | Households that had access to an agricultural plot of land |  |
| Household raised livestock/crops with intent to sell | Households that raise/purchase livestock and/or cultivate crops with the specific intent to sell or resell to earn income.  “Raised livestock” refers to livestock ownership whether livestock was acquired via breeding or purchase. |  |
| **Livestock/Crop Production** |  |  |
| Household raised oxen | Households that raised oxen |  |
| Household raised poultry | Households that raised poultry |  |
| Household raised goats | Households that raised goats |  |
| Household planted crops it made decisions over | Households with a plot of land that planted at least one crop |  |
| **Water, Sanitation, and Hygiene** |  |  |
| Household had handwashing facilitites | Households that had handwashing facilities with water, soap/ash/cleaning agent in the home |  |
| Household correctly treated water | Households that correctly treated water |  |
| Household used improved sanitation facilities | Households that used improved sanitation facilities that were unshared |  |
