# Supplementary material for: Prevalence and risk factors for severe food insecurity and poor food consumption during a drought emergency in Ethiopia
Source: PLOS Glob Public Health. 2025 Sep 11;5(9):e0004636. doi: 10.1371/journal.pgph.0004636 (PMC12425312; doi:10.1371/journal.pgph.0004636)
Supplement: Attachment — Submitted filename: Response to Reviewers.docx [file pgph.0004636.s002.docx]

PLOS Global Public Health Rebuttal Letter

*Prevalence and risk factors for severe food insecurity and poor food consumption during a drought emergency in Ethiopia*

Dear PLOS Global Public Health Editorial and Peer Review Team,

We would like to thank you for your thoughtful and in-depth review of our manuscript. We have incorporated all feedback recommended by the peer review team, and believe these refinements have led to a much stronger version of the manuscript. If there are any further questions or clarifications, please do not hesitate to reach out to Noah Baker ([nbaker17@alumni.jh.edu](mailto:nbaker17@alumni.jh.edu)) or Yunhee Kang ([ykang12@jhu.edu](mailto:ykang12@jhu.edu)). Please find below some important notes that we wanted to ensure are addressed prior to the detailed peer review feedback.

- “Ifaa” is the Ethiopian translation of “RFSA” (Resilience Food Security Activity) and these can be used interchangeably. Both Ifaa and RFSA refer to the USAID-funded Resilience Food Security Activity led by Catholic Relief Services in East Hararghe, Ethiopia. Our study is a secondary data analysis of the baseline survey of this project.
- Regarding data availability, we obtained approval from Catholic Relief Services for the public sharing of this dataset. In accordance with the PLOS Global Public Health Data Availability Policy, we have uploaded the analytic dataset and all code that underlies findings to the recommended data repository, Zenodo. We will link this dataset to the study upon the next steps in this process. (DOI: [10.5281/zenodo.15793020](https://doi.org/10.5281/zenodo.15793020)).

(<https://zenodo.org/records/15793020?token=eyJhbGciOiJIUzUxMiJ9.eyJpZCI6ImJiNTJmYmFlLWY0MTAtNGJhMi04MDAzLTIzMDA3NDUxOWNlNCIsImRhdGEiOnt9LCJyYW5kb20iOiJiM2M0MzkwNWU1OTQ2OGZkODExOTEwYmNhZGNlMmRhNiJ9.HeMgzZwjpPmG0Q3MAKk2wXU3ETwZU4RNE2mzreCafUs6EUt_1xL2KvxIyD6ebJi4sc9CNRj8rbaUnCp5SrU14g>).

- Regarding funding information, the sole funding source should be listed as the United States Agency for International Development Bureau of Humanitarian Assistance.

Further information on data availability and funding is provided in the *Journal Requirements* section below.

We would also like to notify the editorial and peer review team that we found a transcribing error in the original manuscript from the *Severe Food Insecurity* multivariate logistic regression model for the variable “Children Under 5”. This was due to a mislabeling issue. With the detection of this, there is one more statistically significant relationship to note: that households with two or more children were 0.72 times less likely to experience severe food insecurity, exhibiting a similar association as with poor food consumption. This has been appropriately reflected throughout the abstract, manuscript, tables, and figures.

**Original Manuscript Version**

| **Number of CU5 in household** | 4,628 | Crude OR | 95% CI | p | Adjusted OR | 95% CI | p |
| --- | --- | --- | --- | --- | --- | --- | --- |
| Incorrect label: No CU5 in household (Ref):  Correct label: One CU5 in household |  | 1.00 |  |  | 1.00 | . | . |
| Incorrect label: One CU5 in household  Correct label:  No CU5 in household |  | 1.03 | (0.87, 1.22) | 0.749 | 1.14 | (0.94, 1.38) | 0.197 |
| Two or more CU5 in household |  | 0.77 | (0.64, 0.94) | 0.008 | 0.82 | (0.67, 1.01) | 0.059 |

**Corrected Version**

| **Number of CU5 in household** | 4,628 | Crude OR | 95% CI | p | Adjusted OR | 95% CI | p |
| --- | --- | --- | --- | --- | --- | --- | --- |
| No CU5 in household (Ref) |  | 1.00 |  |  | 1.00 | . | . |
| One CU5 in household |  | 0.97 | (0.82, 1.15) | 0.749 | 0.88 | (0.73, 1.07) | 0.197 |
| Two or more CU5 in household |  | 0.75 | (0.64, 0.89) | 0.001 | 0.72 | (0.60, 0.88) | 0.001 |

Please find the remainder of peer review feedback addressed below.

Thank you for your consideration.

Kindest regards,

Noah Baker

MSPH: Johns Hopkins Bloomberg School of Public Health

DO Candidate (Class of 2028): Touro College of Osteopathic Medicine – Harlem

-------------------------------------------------------------------------------------------------------------------------------

**Journal Requirements**

1. *Please provide additional details regarding participant consent. In the ethics statement in the Methods and online submission information, please ensure that you have specified (1) whether consent was informed and (2) what type you obtained (for instance, written or verbal, and if verbal, how it was documented and witnessed). If your study included minors, state whether you obtained consent from parents or guardians. If the need for consent was waived by the ethics committee, please include this information*

“Participants received informed consent during the first module of the Ifaa baseline questionnaire. Participants were informed on the survey purposes, topics, and the person’s rights as a participant, including that their responses will remain confidential and that participation was voluntary. Contact information for the investigators and IRB information was provided. Data collectors conducted the survey in the local language (Oromo) and translated data. Oral consent was provided by participants.”

This verbiage has been inserted in *Informed Consent* from Lines 244-248.

2. *If you are reporting a retrospective study of medical records or archived samples, please ensure that you have discussed whether all data were fully anonymized before you accessed them and/or whether the IRB or ethics committee waived the requirement for informed consent. If patients provided informed written consent to have data from their medical records used in research, please include this information.*

Data was fully anonymous and deidentified at the time the dataset was provided by Catholic Relief Services to the research team. There were no biometrics or medical samples associated with data collection, no medical records were included in this dataset, and retrospective analysis was solely on anonymized survey data. Informed consent was provided to participants, however this is not applicable to this study as this is a secondary data analysis. An exempt determination was provided to the study authors by the Johns Hopkins Bloomberg School of Public Health IRB (FWA: #00000287).

“Data was fully anonymous and deidentified before the dataset was provided to the research team. Oral informed consent was provided to participants. No medical records were included in this research.”

This verbiage has been inserted in *Data Source* from Lines 183-184. IRB information is included in the *Ethics* section of the manuscript.

3. *Your current Financial Disclosure states, “The United States Agency for International Development (USAID) Bureau of Humanitarian Assistance (BHA) provided funding for Ifaa Resilience Food Security Activity (Ifaa/RFSA) to a consortium led by Catholic Relief Services in Ethiopia (720BHA21CA00035). Causal Design was contracted for data collection of the baseline survey for the Ifaa/RFSA project, and to develop an Impact Evaluation under the Implementer-Led Evaluation and Learning (IMPEL) Associate Award funded by USAID BHA, which provided the dataset for this study. The funders had no role in study design, analysis, decision to publish, or preparation of the manuscript”. However, your funding information on the submission form indicates that you received funding from “United States Agency for International Development”. Please indicate by return email the full and correct funding information for your study and confirm the order in which funding contributions should appear. Please be sure to indicate whether the funders played any role in the study design, data collection and analysis, decision to publish, or preparation of the manuscript.*

There was no funding received for execution of this study. This study was a secondary data analysis on the data collected for the baseline evaluation of a project named Resilience Food Security Activity (RFSA/Ifaa) led by Catholic Relief Services and funded by the United States Agency for International Development (USAID) Bureau of Humanitarian Assistance (BHA). USAID BHA should be listed as the sole funding source for this research. As this is a secondary data analysis, the funders had no role in study design, data collection, data analysis, decision to publish, or preparation of the manuscript.

Sole funding source:

United States Agency for International Development Bureau of Humanitarian Assistance

4. *In the online submission form, you indicated that The dataset used in this study is not publicly available, as it is subject to third-party ownership by Catholic Relief Services. Access may be shared upon reasonable request, pending approval from the authors and Catholic Relief Services. All PLOS journals now require all data underlying the findings described in their manuscript to be freely available to other researchers, either*

*1. In a public repository,*

*2. Within the manuscript itself, or*

*3. Uploaded as supplementary information.*

*This policy applies to all data except where public deposition would breach compliance with the protocol approved by your research ethics board. If your data cannot be made publicly available for ethical or legal reasons (e.g., public availability would compromise patient privacy),*

As Catholic Relief Services retains ownership of this dataset, we previously were unable to share data without their approval. We have now received organizational approval from Catholic Relief Services to share this dataset in a data repository. The analytic dataset and code underlying all results are now publicly available on Zenodo at 10.5281/zenodo.15793020. These derive from the Ifaa/RFSA baseline survey (Causal Design, IMPEL. Baseline Study of the Ifaa Resilience Food Security Activity in Ethiopia. Vol. 1. 2022).

Please find the updated data availability statement below.

“**DATA AVAILABILITY:** The dataset and code used to generate the findings of this study are publicly available on Zenodo at 10.5281/zenodo.15793020. These materials were derived from the baseline survey of the USAID-funded Ifaa Resilience Food Security Activity (RFSA), implemented by a consortium led by Catholic Relief Services (“Causal Design, IMPEL. Baseline Study of the Ifaa Resilience Food Security Activity in Ethiopia. Vol. 1. Washington, DC: The Implementer-Led Evaluation & Learning Associate Award (IMPEL); 2022”). For further inquiries, please contact the corresponding author Yunhee Kang (ykang12@jhu.edu) or Noah Baker (nbaker17@alumni.jh.edu).”

5. *Please provide separate figure files in .tif or .eps format*

The prevalence and forest plot figures have been removed from the manuscript and provided as separate files both uploaded in .tiff format.

*Figure 1- Prevalence SFI pFCS.tiff*

This file contains Figure 1 that displays the prevalence of severe food insecurity and poor food consumption.

*Figure 2- SFI pFCS Forest Plot.tiff*

This file contains Figure 2, the forest plot for the multivariate regression with severe food insecurity and poor food consumption.

6. *We notice that your supplementary table is included in the manuscript file. Please remove them and upload them with the file type 'Supporting Information'. Please ensure that each Supporting Information file has a legend listed in the manuscript after the references list*

Supplementary tables have been removed from the manuscript and uploaded as “Supporting Information: Variable Definitions.” A legend for supporting information has been added to the manuscript after the reference list.

*Supplementary Table- Variable Definititions*

**Reviewer #1:**

1. *Comment- 1, it was an interesting tittle your research aim to evaluate PSNP effectiveness, but currently USIAD and the like funding agency were terminated currently. While, this program highly co-correlated with this external donor therefore how about your recommendation according the current status of this donors?*

Editor message indicated that discussing this in the context of terminated USAID funding could be “scope creep.” However, it is critical to mention that new donor sources are required and this has been addressed in Lines 508-510.

2. *Comment-2, since your source of data is secondary data how assures the validity and reliability of the data to get actual finding of the outcome variable.*

The data used in this study were collected by trained enumerators as part of the Ifaa Resilience Food Security Activity (RFSA) baseline study, conducted by Causal Design under the Implementer-Led Evaluation and Learning Award, funded by USAID/BHA. The evaluation employed a **clustered randomized controlled trial (RCT)** design with intervention and control groups covering **4,683 households** across **120 kebeles in Oromia, Ethiopia. The survey was conducted at baseline and no interventions had been implemented at the time of data collection**. There were rigorous randomization, sampling, quality assurance, and data collection procedures. Local data collectors that were fluent with the local language were hired, data collectors were trained in survey administration, and data collection followed structured questionnaires with extensive oversight and field protocols to ensure consistency and data accuracy. The evaluation also included a pre-analysis plan and power calculations to ensure the reliability of outcome estimates. These robust methodological measures in primary data collection implemented by an experienced data collection and evaluation agency, Causal Design, for a consortium led by a leading global humanitarian organization, Catholic Relief Services. The team employed a standard randomized controlled design with data collectors that were trained in the data collection procedures and protocols support the validity and reliability of the secondary data used in the study. Please find below an excerpt from the baseline evaluation regarding data collection activities for the baseline Ifaa survey.

“The data collection activities occurred during May and June 2022, coinciding with the lean season. Researchers conducted the training during the first 6 days of the data collection activities, and a 2-day practical piloting at the field level followed the training. Teams conducted the pilot testing in Woreda Kimbibit in the North Shewa zone of the Oromia region. For the data collection activities, 30 enumerators (eight women and 22 men) and six supervisors were deployed. The enumerators were divided into six teams. Each team was assigned a supervisor and was assigned to survey 1–2 woredas. To ensure quality data, the teams performed a series of activities during and after data collection. The data quality assurance undertaken during the data collection included: office-level phone call-backs and a 10% back-check on the interviewed households. Additionally, the research team undertook high-frequency checks regularly.” ^[1]^

“The sample size was based off power calculations and data collection consisted of strict inclusion criteria, requiring households to be participants in the PSNP program, and one women/girl of reproductive age (WRA) (15-49 years of age) must reside in the household. Catholic Relief Services collaborated with local authorities and PSNP to enumerate PSNP households with women/girls of reproductive age within the target woredas of the Ifaa project”

Lines 165-168

“The respondent to the survey was a WRA that resided in the randomly selected household. In cases where multiple WRA resided within the household, the respondent was selected either randomly, or purposively in the case that one WRA was more knowledgeable about the answers to the questions being asked. The selected WRA completed the Women’s health, nutritional status, dietary diversity, and family planning modules and provided information on individual and household sociodemographics, economic activity, crop/livestock production, and Water, Sanitation, and Hygiene (WASH) materials/practices. The age groupings for WRA indicate the age groups of the WRA respondent. Data was fully anonymous and deidentified before the dataset was provided to the research team. Oral informed consent was provided to participants. No medical records were included in this research.”

Lines 177-184.

3. *Comment-3, the sample size of you used for food insecurity and food consumption is quite different why this difference happens?*

The sample size between food insecurity and food consumption differs as a result of missing data within the sample. The inclusion criteria of the multivariate logistic regression required that each individual within the sample must have answered the questions to all other variables assessed in the regression analysis for each outcome. Those missing data from either the food insecurity or food consumption sample were excluded from further analysis with the respective outcome to ensure appropriate bivariate comparison. This is consistent with the complete case analysis method for handling missing data. There is an overall difference of respectively 67 individuals for the multivariate regression samples between the outcomes of severe food insecurity and poor food consumption, or a 1.55% difference. This has been noted in the *Limitations* section (Line 521-524).

4. *Comment-4, productive safety net program is not much productive effectively even highly supported with donor like USAID and other agencies. In addition USAID currently it was terminated therefore what recommendation you plan for Ethiopian government especially on local intervention that reduce food insecurity.*

We agree with reviewers comment on how critical it is to still implement local interventions to improve food security, even in circumstances of more limited funding. In the context of the changing financial landscape with the termination of USAID funding, based on the consolidation of evidence through data analysis and contemporary literature we have provided suggestions to address this and mitigate food insecurity in drought prone regions. There must be a coordinated response at the national level that collaborates with local and global aagencies to determine the most suitable methods to address this change in financial structure.

To avoid potential scope creep as suggested by the PLOS editorial office, this has been addressed via the following:

“Lastly, in the context of the changing financial landscape with the termination of USAID funding, there must be a coordinated response at the national level that collaborates with local and global agencies to identify and mobilize alternative funding sources.” (Lines 508-510)

***Reviewer #2:***

*Introduction*

1. *you use a lot of abbreviations. If they are not reintroduced, don't add them. Specifically line 114 IDP.*

We are appreciate this being mentioned, and abbreviations that are not reintroduced were removed from the manuscript (including IDP, FAO, FEWS, OCHA, SDG, RFSA, ESSWA) . Unnecessary abbreviations that were not consistent throughout the manuscript such as HH for households have been removed and replaced with “household/s.”

2. *line 117 - why Russia and Ukraine*

Russia and Ukraine was mentioned in the introduction because of these findings from the initial literature review: “The ripple effect of the war in Ukraine is set to exacerbate the food security crisis in Ethiopia, where over three quarters of WFP’s and the government’s wheat – the staple cereal - comes from Ukraine or Russia. The crisis in Ukraine threatens to push the cost of wheat and fertilizer beyond the means of millions of farmers, threatening this year’s harvest.” (World Food Programme. Conflict, climate and soaring food prices push Ethiopia further into hunger while WFP funding runs out [Internet]. WFP; 2022 Jun 23 [cited 2025 Apr 10]. Available from: <https://www.wfp.org/news/conflict-climate-and-soaring-food-prices-push-ethiopia-further-hunger-while-wfp-funding-runs>).

While the Russia-Ukraine war likely did have an impact on food security in Ethiopia, it may not be quantifiable and deviates away from the focus of this study. After revisiting this we believe that this does not need to be mentioned in this study and this wording has been removed.

Line 123-124

3. *- line 118 - are you talking about chronic or acute severe food insecurity?*

Protection cluster report does not specify chronic or acute food insecurity. However it is phrased as, “Severe food insecurity due to recurrent drought, flood damage, and pests,” and therefore can be inferred as chronic severe food insecurity due to the word recurrent in reference to drought, flood, and pests. (UNHCR Protection Cluster. Protection Cluster Ethiopia: Monthly Protection Overview – September 2023 [Internet]. ReliefWeb; 2023 Oct 9 [cited 2025 Apr 10].) Regarding the manuscript, we have kept this wording the same as is described in the UNHCR Protection Cluster Report.

We added the word chronic in reference to drought, flood, and pests, but have kept the wording on “severe food insecurity” as it reflects the described outcome in the UNHCR report. (Line 124-127)

4. *line 120 - cholera: again, is it chronic or acute? and how does that impact food insecurity?*

Cholera reference was included to provide context for the nutritional susceptibility of this population and was included within the UNHCR Protection Cluster report. “Cholera cases reported in Kersa, Haromaya, Kurfa Cheleworedas of East Hararghe and Gumbi Bordede of West Hararghe.” (UNHCR Protection Cluster. Protection Cluster Ethiopia: Monthly Protection Overview – September 2023 [Internet]. ReliefWeb; 2023 Oct 9 [cited 2025 Apr 10].) The cholera references have been removed from the manuscript, as they are not directly relevant to the topic being assessed- food security and food consumption in drought, and we feel that it does not benefit the study overall.

5. *you need more context, and indicator for lines 124-128. Frame the problem, provide prevalence estimates.*

Context to the problem has been added and prevalence estimates have been included according to the Sustainable Development Report data points.

“Although the Sustainable Development Goals aim to reach zero hunger in Ethiopia by 2030, the 2023 report indicated “major challenges” still remain to achieve this. While progress has been made in child wasting (6.8%), recurrent drought and conflict, along with socioeconomic barriers, have contributed to the major challenges to reduce the magnitude of undernutrition (21.9%) and child stunting (36.8%).” (Lines 131-134)

6. *Lines 128-130: I suggest you change the language to say you want to identify a solution*

Language has been altered in accordance with this recommendation.

Original: “The persistent gap between food aid requirements and available assistance primarily experienced by PSNP households reinforces the need for research on PSNP households to develop tailored interventions and support progress toward the SDG of zero hunger.”

Proposed change: The persistent gap between food aid requirements and available assistance primarily experienced by PSNP households reinforces the need to identify a solution. (Line 136-137)

7. *lines 135 - you mention it here (I wondered why) and once in the discussion that the data were collected during Ramadan or another time of fasting. 1. explain that clearly. 2. how is that related to food insecurity? I can see food consumption and I think that the literature (informal at least) suggest that food consumption patterns change but maybe not quantity. So, that needs to be clarified and contextualized.*

We are grateful for this note by the peer review team, as it was our original impression that data collection overlapped with fasting periods such as Ramadan. Ramadan occurred from Friday, April 1, 2022 – Sunday, May 1, 2022, and data collection for the Ifaa baseline evaluation occurred between **May and July 2022. Since May 1, 2022 was a Sunday, data collection began after and continued throughout June. Data collection did not overlap with Ramadan/fasting periods. Additionally, the East Hararghe Zone is predominantly Muslim, with low members of the Christian Orthodox religion that abstain from animal source foods on Wednesdays and Fridays. Therefore, fasting is not expected to have impacted the data. This was an inconsistency that we did not initially recognize and thank the peer review team for highlighting this. All references to fasting/Ramadan have been removed from the manuscript.**

*Methods*

1. *Please provide additional details on who completed the survey. Was it the primary caregiver who was a WRA? What if there were many WRA in a household. Your results have multiple age groupings for WRA - were they the primary WRA and completed the survey or were they living in the household?*

The survey respondent was a woman/girl of reproductive age that resided within the selected household. The WRA respondent provided information on individual and household characteristics. The households were randomly selected from the sampling frame provided by Catholic Relief Services. The age groupings for WRA indicate the age groups of the WRA respondent. For within household selection for households with more than one WRA, the evaluation team randomly or purposively selected one target individual for each outcome. This was because interviewing every eligible individual in the household is time consuming and costly for both data collection and the participant. Additionally, the outcomes would be highly correlated between WRA members of the same household. Therefore, information collected is representative of the WRA respondent’s household.

“The survey respondent was a WRA that resided in the randomly selected household. In cases where multiple WRA resided within the household, the respondent was selected either randomly, or purposively if one WRA was more knowledgeable about the answers to the questions being asked. The selected WRA completed the Women’s health, nutritional status, dietary diversity, and family planning modules and provided information on individual and household sociodemographics, economic activity, crop/livestock production, and Water, Sanitation, and Hygiene (WASH). Age groupings indicate the age groups of the WRA respondent. Data was fully anonymized and deidentified before it was provided to the research team. Participants provided oral informed consent. No medical records were included in this research.”

These details have been inserted in Lines 177-184.

2. *covariates: more definitions are needed.*

Covariate definitions have been refined based on information from the baseline survey to appropriately describe the characteristic of the woman/girl of reproductive age and/or household that is being assessed. Other than age group and number of CU5 in the household which are categorical variables, the reference for each variable is the counterfactual.

Lines 208-225

The covariate supplementary table has been uploaded as a supplementary file as per the instructions by the PLOS editorial team.

| **Variables** | **Definitions** |  |
| --- | --- | --- |
|  |  |  |
| **Age Group** | Age group of the woman/girl of reproductive age (WRA) (15-49 years) that responded to the survey |  |
| WRA 20-29: Reference Group | Households where the woman/girl of reproductive age survey respondent was between 20 and 29 years of age. |  |
| WRA 15-19 | Households where the woman/girl of reproductive age survey respondent was between 15 and 19 years of age. |  |
| WRA 30-49 | Households where the woman/girl of reproductive age survey respondent was between 30 and 49 years of age. |  |
| **Number of CU5 in household** | The number of children under 5 years of age that resided within the household that was surveyed. |  |
| No CU5 in household: Reference Group | Households that did not have any children under 5 years of age residing within them. |  |
| One CU5 in household | Household that had one child under 5 years of age residing within it. |  |
| Two or more CU5 in household | Households that had 2 or more children residing within them. |  |
| **Demographics** |  |  |
| WRA Currently Pregnant | Households where the woman/girl of reproductive age respondent was pregnant at the time of data collection. |  |
| WRA History of Pregnancy | Households where the woman/girl of reproductive age respondent had a history of pregnancy |  |
| WRA Marital Status: Married | Households where the woman/girl of reproductive age respondent was married. |  |
| **Educational Level** |  |  |
| WRA No school | Households where the woman/girl of reproductive age survey respondent did not receive any formal education. |  |
| Household Head No school | Households where the household head did not receive any formal education  Household head refers to either the household’s woman/girl of reproductive age respondent if the woman/girl of reproductive age was the household head, or the individual the woman/girl of reproductive age respondent considered to be head of the household. The woman/girl of reproductive age respondent provided this information regardless of who was the head of household. |  |
| **Economic Activity** |  |  |
| WRA performed cash earning work last 12 months | Households where the woman/girl of reproductive age survey respondent performed cash-earning work in the past 12 months |  |
| Household saved money | Households that saved money |  |
| Household had daily per capita food consumption costs <1.61 USD | Households that consumed less than 1.61 USD of food per day on average. Indicator of food poverty based on the 2011 adjusted poverty line computed via consumer price index. |  |
| Household used financial services | Households that either used agricultural credit, had savings, and/or used crop insurance |  |
| Household had access to a plot of land | Households that had access to an agricultural plot of land |  |
| Household raised livestock/crops with intent to sell | Households that raise/purchase livestock and/or cultivate crops with the specific intent to sell or resell to earn income.  “Raised livestock” refers to livestock ownership whether livestock was acquired via breeding or purchase. |  |
| **Livestock/Crop Production** |  |  |
| Household raised oxen | Households that raised oxen |  |
| Household raised poultry | Households that raised poultry |  |
| Household raised goats | Households that raised goats |  |
| Household planted crops it made decisions over | Households with a plot of land that planted at least one crop |  |
| **WASH** |  |  |
| Household had handwashing facilitites | Households that had handwashing facilities with water, soap/ash/cleaning agent in the home |  |
| Household correctly treated water | Households that correctly treated water |  |
| Household used improved sanitation facilities | Households that used improved sanitation facilities that were unshared |  |

3. *Financial services, for example, is that using a bank? Later in the results you refer to a loan. That is a big difference and helpful to know up from.*

A household uses financial services if at least one of the following is satisfied: (i) the household uses agricultural credit, (ii) the household has savings, or (iii) the household uses crop insurance. This has been noted in Line 218.

4. *Also, your dichotomous variables are not 'opposites'. For example, #5 raised livestock/cultivated crops with intent to sell - the opposite is raised with no intent to sell NOT did not raise. Same with #7. I think it is a definitional issues so please provide more context.*

This definitional issue arises because in the *Covariates* section we mentioned that the opposite of raised or buy livestock/cultivated crops with intent to sell was-“did not raise”; when the counterfactual would actually be- “did not raise with intent to sell.” Also please note that “raised livestock” also includes households that purchased livestock. This has been accurately reflected in the *Covariates* section (Lines 219-221). The variable has also been appropriately defined in the *Supplementary Table:* *Variable Definitions.* The peer review team is also referring to the variables raised oxen, poultry, and goats, which are three separate variables, and each has the counterfactual now mentioned in the *Covariates* section (Line 221)We thank the peer review team for the notice, and this has been accurately reflected in the manuscript. We also want to note that this definitional issue does not change the conclusion or recommendations, as these refer to prioritizing livestock production overall and upgrading the livestock market, which is supported by evidence.

Definition: Households that cultivate crops or raise/buy livestock with the specific intent to sell or resell to earn income

*Results*

1. *Tables: If you chose to use the FI sample, you don't need table 2. If you want to show there are no differences, combine them into one table and show statistical testing for each variable showing no difference*.

We appreciate the suggestion by the review team and this has been implemented. Since the food insecurity sample was used for descriptive analysis due to its larger sample size, descriptive analysis was not performed on the poor food consumption sample. Table 2 has therefore been incorporated into the Original Table 1 (Now Table 2), and a two sample proportions z-test was conducted to confirm that there are no significant differences between the two samples.

2. *Table - is the variabel household head no school the survey taker or the male head of household? Who is that?*

The variable “Household head no school” refers to either the household’s WRA survey respondent or the individual that was considered head of the household. The WRA respondent provided this information regardless of who was considered head of household. This has been expressed in the *Supplementary Table- Variable Definitions.*

3. *Line 250: Thiis an example of the WRA question. You state 'households with WRA aged 15-19...' Does that mean the personal who took the survey? As 'primary WRA' or is it a WRA of that age living in the household. Clarify the sentence. If you mean 'respondent' rather than 'household with', please fix.*

This means that the WRA respondent of the household was aged 15-19. The WRA took the survey on behalf of their household. This verbiage has been adjusted in accordance with these suggestions.

“The distribution of age groups indicated that 60.2% of the household’s WRA respondents were 30-49 years, 16.36% were 15-19 years, and 23.44% were 20-29 years.” (Lines 253-255)

4. *I have to say, there were many variables with unexpected findings. Households with more children under the age of 5 had less food insecurity. Also, WRA 15-19 was associated with higher food insecurity but better food consumption. Same with history of pregnancy, pregnancy, and raising crops. I think your discussion touched on some of that but it is really unexpected.*

We would like to thank the peer review team for bringing this up, as these unexpected associations are key findings within this study, and we believe these findings increase the importance that this study is shared in literature. We conducted a thorough assessment of model validity by evaluating the consistency, directionality, magnitude, and precision of adjusted odds ratios across both outcomes. We also tested if the model accounted for potential confounding through multivariate logistic regression, which was confirmed as exemplified by the independence of associations. A full explanation of the statistical rationale and regression model validity is included below for the reference of the peer review team. The interpretive rationale is described in the manuscript throughout the *Discussion* section with estimated line numbers included. Additionally, a study conducted in West Africa by Jonathan Lane et al found that a “significant share of the population is severely food insecure according to the FIES, despite having an FCS that is far above the threshold for poor or borderline consumption or expenditure that is far above the threshold for monetary poverty.”^[2]^ A screenshot of this finding from the study is included below, along with the study reference listed. This has been incorporated into the manuscript, and reflects the nuances between the classification of food security status with Food Insecurity Experience Scale and measure of Food Consumption Score, as households with adequate food consumption over a week period may still be categorized as severely food insecure based on their previous 12 months of experience.

Statistical Validity

Although households with WRA who were currently pregnant, history of pregnancy, aged 15–19 years, and households that planted crops they make decisions over had higher odds of severe food insecurity, but lower odds of poor food consumption, these associations were robust in direction and magnitude across both adjusted models. With poor FCS, the statistical relationship displays narrow confidence intervals (95% CI Maximum Observable Difference = 0.44), indicating high precision of estimates and 95% likelihood that the true estimates lie within the confidence interval. For severe food insecurity, the confidence intervals are slightly wider, but all except “History of Pregnancy (95% CI Width = 1.57)” are within ± 0.98. However, this is the largest magnitude seen in the study outside of the association of Daily Per Capita Food Consumption Costs with poor FCS, which could have led to the slightly lower precision in adjusted odds ratio estimate for “History of Pregnancy.” Importantly, the positive association of “History of Pregnancy” with severe food insecurity is still significant at 99.6% confidence level. The other three variables positively associated with severe food insecurity have a narrower 95% Confidence Interval, with a Maximum Observable Difference of 0.98, which is still relatively narrow. This consistency indicates that the model was well specified, appropriately adjusted for confounders, and capable of isolating the independent effect of each variable on its respective outcome.

| **Variable** | **SFI AOR [95% CI]** | **SFI p-value** | **Poor FCS AOR [95% CI]** | **Poor FCS p-value** |
| --- | --- | --- | --- | --- |
| **Currently Pregnant** | 1.52 [1.17, 1.96] | <0.002 | 0.57 [0.47, 0.70] | <0.001 |
| **History of Pregnancy** | 3.46 [2.76, 4.33] | <0.001 | 0.70 [0.55, 0.89] | 0.004 |
| **Aged 15–19 Years** | 1.79 [1.36, 2.34] | <0.001 | 0.59 [0.46, 0.76] | <0.001 |
| **Planted Crops** | 1.67 [1.31, 2.13] | <0.001 | 0.57 [0.44, 0.75] | <0.001 |

Furthermore, the multivariate logistic regression model appropriately adjusts for confounding by controlling for the statistical impact covariates with overlapping influence on both food insecurity and food consumption outcomes. Notably, the variable for history of pregnancy demonstrated consistent directionality in both models and showed strong associations with each outcome. Given that 62% of WRA aged 15-19 had a history of pregnancy, this prevalence supports the model’s ability to detect statistical associations while controlling for correlated variables such as age and pregnancy status. Importantly, the model isolates the independent effect of each variable. For example, 65% of the 15–19 year age group had poor food consumption, which is a lower proportion than the 70% and 72% observed in the 20–29 and 30–49 year groups, respectively. While these sample size differences may have contributed to variation in significance levels, this indicates the model accurately detected a significant difference between age groups. The direction and strength of associations remained stable across the regression, further validating that the model is correctly accounting for confounding and yielding reliable estimates of independent effects.

An additional variable that emphasizes the validity of this statistical model is Daily Per Capita Food Consumption Costs <1.61USD. This variable is responsible for two out of the three strongest associations with severe food insecurity and poor food consumption score and exhibits associations that are similar in both magnitude and directionality for each outcome. A similar confirmation can be used with the variables: Raised Oxen and Raised Livestock/Cultivated Crops with the intent to sell, as these variables exhibit strongly protective associations that are similar in both magnitude, directionality, and precision (based on 95% CI) for both severe food insecurity and poor food consumption.

The same statistical justification exists for households that planted crops they make decisions over, however, we believe it is key to interpret this in context with the weighting of the food consumption score measure. While planting crops may not facilitate food security particularly in drought prone settings where agricultural production is limited, it provides a source of fruits, vegetables, nuts/seeds, and roots/tubers/pulses. These are food groups that are commonly incorporated into meals, and importantly, many are extremely nutrient dense. Since food consumption score weights the nutrient value of foods consumed and households that plant these crops have eased access to these supplemental food groups, they can more easily include these in their diet. The additions in vegetables, fruits, nuts/seeds, roots/tubers/pulses could lead to improved food consumption scores because of the additional food groups pulses that are now added into the FCS calculation. Access to small amounts of these food groups from planting crops that these households own can theoretically increase FCS in times of sufficient crop yield, even in households that are categorized as severely food insecure.

Lines 352-385: Rationale and interpretation of households with WRA aged 15-19, currently pregnant, and/or history of pregnancy.

**Crops**

Lines 433-437

5. *Table 3 has a ? in 'daily per capita...'*

This has been removed and is not present in our current manuscript file.

6. *I was unclear about what you were saying in lines 259-261*

This refers to explanation of the relationship between the variables: 1. Planted crops they make decisions over and 2. Raised livestock/cultivated crops with the intent to sell. The far majority of the overall sample planted crops (91%), and 92.48% of severely food insecure households planted crops. Therefore the increased risk of severe food insecurity with planting crops is likely driven from the proportion of households that only planted crops, as opposed to those that also owned livestock. Considering nearly all households planted at least one crop, the protective association between raised livestock/cultivated crops to sell and severe food insecurity was likely driven from the proportion of households that either solely raised livestock or raised livestock and planted crops. With recurrent drought it follows that planting crops may not improve food security since there is low precipitation and harsh growing conditions, and therefore livestock in these settings would have a greater positive impact on food security. This wording has been adjusted to more appropriately reflect that or the wording can be removed.

“Although 91% of all households planted crops, high levels of severe food insecurity persisted. Among severely food insecure households, 92.48% planted crops, suggesting this risk factor was driven primarily from by households that planted crops and did not raise livestock. As only 34.98% of severely food insecure households raised target livestock, this supports the observed protective association with raising livestock/cultivating crops to sell. This protective effect is likely attributable to households that either solely raised livestock, or engaged in livestock and crop production for market-oriented purposes.” (Lines 285-290)

Discussion:

1. *starting at 344- what are some reasons*

The *Sociodemographic Characteristics* section (starting at Line 350) has been reworded and rearranged in accordance with peer review recommendations. Statistical rationalities along with interpretations based on evidence from data analysis and literature have been concretely incorporated in this section. Additionally there is an in-depth explanation of the results and inverse associations between WRA pregnancy, history of pregnancy, WRA age 15-19, and planting crops in their respective *Discussion* sections.

2. *Economic activity: I was thinking a lot about directionality, which you did not talk about. It is possible that people worked because they were food insecure, for example. That is true for many of the indicators. Or, they were forced to do income generating activities because of the drought.*

The directionality is extremely important to highlight, and we are very glad that this was noted by the peer review team. The counterfactual has now been discussed in this section, along with calls for future research into this topic, and tying it in with the nuances between food security status and food consumption. (Lines 400-411).

3. *Lines 360+ - I think you are refering to immediate demand versus long term stability - living from "check-to-check" - is that right?*

This is a correct interpretation, but in the context of utilizing financial resources towards food acquisition vs. saving money to increase overall financial capacity (and how saving may not be feasible in drought emergency settings which may require the former).

Lines 400-407

4. *374 - the use of credit could be because of the food insecurity*

This is a very thoughtful suggestion and has been incorporated into the manuscript. This also highlights the need for further research into the directionality of these relationships and points for intervention.

Lines 415-416

5. *Line 385 - extra word 'above'*
   The word above no longer appears in the manuscript as confirmed via CTRL-F. The word “above” has spontaneously been inserted next to reference numbers upon our altering of the endnote reference formatting, and this has been addressed throughout the manuscript.
6. *The water, sanitation and hygiene section was really limited. And, your table showed that having handwashing materials was associate with higher food insecurity. What do you think about that?*

The relationship between food security and WASH does provide some interesting insights, and we are thankful for this being mentioned by the peer review team. We now believe that we have appropriately reflected our inferences based on the relationships between WASH and food security/consumption. As the statistical relationship between food security, food consumption, and WASH indicators has not been thoroughly explored in literature, and with only 2.36% of the sample consisted of households with handwashing materials, we did not initially prioritize WASH in the discussion section. However, using improved sanitation exhibited one of the strongest protective relationships for severe food insecurity and poor food consumption. We have analyzed this further and highlighted the significance, and we believe improved sanitation is indicative of households with greater financial resources. We have also inserted our interpretation of handwashing materials, which could be a result of households redirecting financial resources towards WASH items as opposed to food (i.e. potentially because of child diarrhea or other gastrointestinal infection prevalence). While limited on references, this does call for increased future research on this topic, which is included in the discussion section as well, along with integrating WASH with food security programming.

Lines 477-487

7. *lines 464 - I don't know if that is true or not that there are unique features due to fasting. It doesn't align with your argument of long term drought conditions*

Fasting considerations have been removed from the manuscript as Ramadan did not overlap with the data collection. Please see response to Reviewer 2 comment 7 in the Introduction section.

-------------------------------------------------------------------------------------------------------------------------------

We would like to thank the editorial and peer review team again for the opportunity to improve this manuscript through your thoughtful feedback. We have found the feedback to be extremely insightful, and believe these revisions have significantly strengthened the manuscript. Please do not hesitate to reach out with any further questions, concerns, or comments. We look forward to the next steps in this process.

Thank you again,

Noah Baker

1. Causal Design, IMPEL. Baseline Study of the Ifaa Resilience Food Security Activity in Ethiopia. Vol. 1. 2022
2. Lain, Jonathan, Sharad Tandon, and Tara Vishwanath. “How Much Does the Food Insecurity Experience Scale Overlap with Poor Food Consumption and Monetary Poverty? Evidence from West Africa.” *The World Bank Economic Review* 38, no. 2 (April 29, 2024): 422–42. <https://doi.org/10.1093/wber/lhad031>.
