# Supplementary material for: Prevalence and risk factors for severe food insecurity and poor food consumption during a drought emergency in Ethiopia
Source: PLOS Glob Public Health. 2025 Sep 11;5(9):e0004636. doi: 10.1371/journal.pgph.0004636 (PMC12425312; doi:10.1371/journal.pgph.0004636)
Supplement: Attachment — Submitted filename: plos Food insecurity comment.docx [file pgph.0004636.s003.docx]

Tittle - Prevalence and risk factors for severe food insecurity and poor food consumption during a drought emergency in Ethiopia

Overall aspect of this manuscript so nice. This research asses retrospectively in this case how how about the validation of data to infer general population during a drought emergency of Ethiopia. Since the data source is secondary data how refine or filter the data which was incomplete

Otherwise it was very interesting overall work
